# Micro-CT-Based Experimental Study on the 3D Structure–Electrical Conductivity Relationship of Metal-Coated Woven Fabrics

**DOI:** 10.3390/ma19153244

**Published:** 2026-07-31

**Authors:** Magdalena Tokarska, Adam K. Puszkarz, Marcin Makówka

**Affiliations:** 1 Faculty of Textiles and Design, Institute of Architecture of Textiles, Lodz University of Technology, 116 Zeromskiego St., 90-543 Lodz, Poland; 2 Faculty of Textiles and Design, Textile Institute, Lodz University of Technology, 116 Zeromskiego St., 90-543 Lodz, Poland; adam.puszkarz@p.lodz.pl; 3 Faculty of Mechanical Engineering, Institute of Materials Science and Engineering, Lodz University of Technology, 1/15 Stefanowskiego St., 90-924 Lodz, Poland; marcin.makowka@p.lodz.pl

**Keywords:** micro-CT, 3D woven fabric geometry, metal coating, electrical conductivity, electrical anisotropy

## Abstract

**Highlights:**

- X-ray micro-computed tomography enables characterization of the structure–conductivity relationship in metal-coated fabrics.
- Fabric pore structure shows a stronger relationship with electrical conductivity than coating thickness.
- Increase in pore sizes in woven fabrics may increase in-plane electrical anisotropy.
- Extreme electrical resistance values occur along the warp and weft directions.
- Electrical anisotropy should be considered in metal-coated woven fabric design.
- Woven fabric structure analysis supports conductivity-oriented fabric design.

**Abstract:**

The investigated materials were metal-coated woven fabrics. X-ray micro-computed tomography was employed to characterize the fabrics, including their coating, yarn, and pore structures. Electrical resistance was measured using a four-electrode configuration inspired by the van der Pauw method. The obtained results revealed strong relationships between structural parameters and electrical properties of fabrics. Multidirectional resistance measurements confirmed continuous electrical conduction and low resistance values. Correlation analysis revealed that a thicker metal coating on the fabric surface may be associated with increased anisotropy of the electrical properties, with a correlation coefficient of 0.772 at the 0.05 significance level. The results suggest that an increase in pore sizes (mean and median) may be associated with higher resistance values (correlation coefficients ranged from 0.839 to 0.941 at the 0.05 significance level), potentially contributing to changes in the distribution of conductive regions within the fabric. An increase in pore sizes (median) may be associated with greater dimensional irregularity, which in turn may be related to increased direction-dependent variability in the electrical response, reflecting higher electrical anisotropy within the sample plane (correlation coefficient of 0.958 at the 0.05 significance level).

## 1. Introduction

Electroconductive fabrics are mainly used as technical textiles serving as sensors, shielding materials, heating elements, signal pathways, leads, and connectors [1,2,3,4,5]. Due to their flexibility and low weight, the fabrics can be inserted into the driver’s seat for heating purposes [6,7], used in protective clothing as textile signal lines [8,9], or serve as an integral element of an orthosis intended for electrostimulation [10,11]. Technologies such as weaving, knitting, and embroidery can produce textile products that can conduct electricity [11,12,13,14]. Complex structures characterize these fabrics, and obtaining specific electroconductive properties requires considering many factors, e.g., the content of the electroconductive yarns in the structure, fabric weave, density, and thickness [15,16,17]. Other technologies are also employed for surface modification and the production of electroconductive products. Conductive paste or ink printing on non-conductive fabric can also be used for electroconductive materials production [18,19,20]. Printing electroconductive paste is well-suited for designing electrical circuits; however, selecting the paste thickness so that the path has low resistance while also being resistant to washing and bending is undoubtedly a challenge [18]. Conductive inks printed on textile substrates are vulnerable to cracking because of the substrate’s deformable and porous structure [19]. The fabric substrate’s smallest pore size and roughness are essential from the point of view of higher printing precision and lower electrical resistance of screen-printed conductive lines [20]. The non-uniformity of the applied conductive paste layer causes the textile materials to be relatively thick compared, for example, to metalized fabrics. A rough surface might increase friction and wear, affecting the material’s longevity. It also causes the nonuniform flow of electric current in the fabric. Chemical and physical vapor deposition enables material surface modification with thin metal coatings to obtain thin electroconductive fabrics [14,21,22]. These fabrics are characterized by excellent electrical conductivity. Metal deposition processes are applied to fabrics with high yarn density, thereby making the material thinner and the surface smoother. However, research indicates surface roughness in metalized fabrics [23], as the thin metallic coating maps the sample topography. Electrical in-plane anisotropy is observed in these materials [23,24].

In general, woven fabrics are anisotropic rather than isotropic materials from the point of view of thermal, mechanical, and electrical properties [23,24,25,26,27,28,29,30]. Surface topography significantly affects the electrical resistance of the fabric. The electric potential and current density distributions depend on how the yarns are interlaced and the electric current input directions. The contact points between yarns should be considered because they play a significant role in the fabric [31,32,33,34,35]. High contact resistance between components (fibers, yarns) can lead to hotspots, potentially causing user discomfort, skin burns, or damaging fabric-based devices, such as sensors [36,37]. To explain the role of contact between elements in textile materials, woven structures made of metal-coated fabric strips were designed [33]. It was found that the number of contacts between strips, directly resulting from structure density, affects the electroconductive properties of these structures. Textile composites containing carbon fibers were also investigated [34]. It was noticed that when conductive fibers break, electrical current flows more easily through the contact points in the transverse direction because of the relatively low resistivity in this direction. The small gaps between the contacting points act as capacitors and allow some displacement current to flow [38,39]. Impurities on the material surface, nonuniform fabric structure features, and a uniformly distributed electroconductive coating can also alter current conduction [35,40]. It was found that the floating cover firmness factor and the conductive yarn percentage significantly affect the contact quality between the measuring electrode and the textile substrate, which influences the electrical resistance measurement results [41]. The cover firmness factor determines the floating length of conductive yarns in the fabric weave. The higher the factor, the greater the number of interlacing points between the warp and weft yarns and the higher the electrical surface resistivity. The higher the percentage of electrically conductive fibers/yarns in the fabric, the lower the resistivity. Fabric is not an ideal periodic woven structure, which affects the electroconductive properties of the fabric after surface modification. It was found that the smaller the area of the metal-coated woven fabric, the fewer disturbances, and the decrease in the electrical in-plane anisotropy [27].

The electrical conductivity of metalized woven fabrics results from the outer metal layer deposited on the non-conductive textile surface, whose structure acts primarily as a geometric substrate for this coating. These textile materials are characterized by dimensional stability, high structural density, low elasticity, tear resistance, and greater creasing strength than knitted fabrics. Due to their thinness, they are light and smooth to the touch. Therefore, these fabrics would be expected to have isotropic electrical properties. Meanwhile, electrical anisotropy is often observed in these textile materials and needs to be identified. These fabrics generate an electrical signal; therefore, how this object is connected to other components of the measurement system or device affects the system’s operation. From the perspective of designing such fabrics, identifying the fabric structure geometry and specifying the parameters that affect electrical resistance are crucial. Optical methods operating on two-dimensional image projection enable mapping of pore size, shape, and distribution in woven fabrics [42,43,44]. The analysis of 2D images enhanced by digital image processing techniques requires making assumptions, especially for a textile object. It is assumed that yarns are uniform along their length and equally spaced in the fabric; there is no yarn fattening. Scanning electron microscopy (SEM) is also a tool for sample surface evaluation. Surface topography and morphology of a fabric can be obtained, but not the internal sample structure [45,46]. SEM is limited to two dimensions because it records only the intensity of emitted electrons as a function of the beam position. The structure characterization of electroconductive woven fabrics requires a transition from 2D imaging techniques to advanced 3D analysis methods. High-resolution micro-computed tomography (micro-CT) empowers 3D geometric reconstruction and analysis of the woven fabric structure. The effect of three different surface modifications (flocking, layer-by-layer, and screen printing) on the electroconductive properties of woven fabrics of different raw material compositions (cotton, viscose, polyester) was examined using microtomography to obtain characteristics of the structures, and to assess the effect of the three types of applied surface modifications on the structure of the woven fabrics [47]. X-ray tomography, alongside optical microscopy and scanning electron microscopy, was used for testing polymer composites reinforced with electrically and thermally conductive carbon fiber fabric based on nanocarbons [48]. The goal was to investigate the multilayer, realistic structure, including nanofiller dispersion, internal defects (larger than 0.7 μm), and carbon fiber architecture. In another study [49], the surface topography was characterized using field emission scanning electron microscopy (FESEM) and atomic force microscopy (AFM), and tomography of the composite was realized through a micro-CT scan in studies on developing waterproof, breathable, conductive cotton fabric.

Although micro-CT has been successfully applied to the characterization of a wide variety of materials, its application to the quantitative assessment of pore and yarn size distribution and coating in metalized woven fabrics has not yet been reported in the literature, to the best of the authors’ knowledge.

## 2. Materials

Metalized woven fabrics manufactured by Shieldex^®^ (Bremen, Germany) were chosen as the materials to be evaluated for their electrical properties. The polyester (PES) and polyamide (PA) fabric surfaces were modified with silver, copper, tin, and nickel coatings. For the chosen fabrics, protective acrylic (CR) and conductive polyurethane (PU) coatings were applied without affecting the electroconductive properties. The fabric characteristics declared by the manufacturer are given in Table 1.

**Figure 1:**
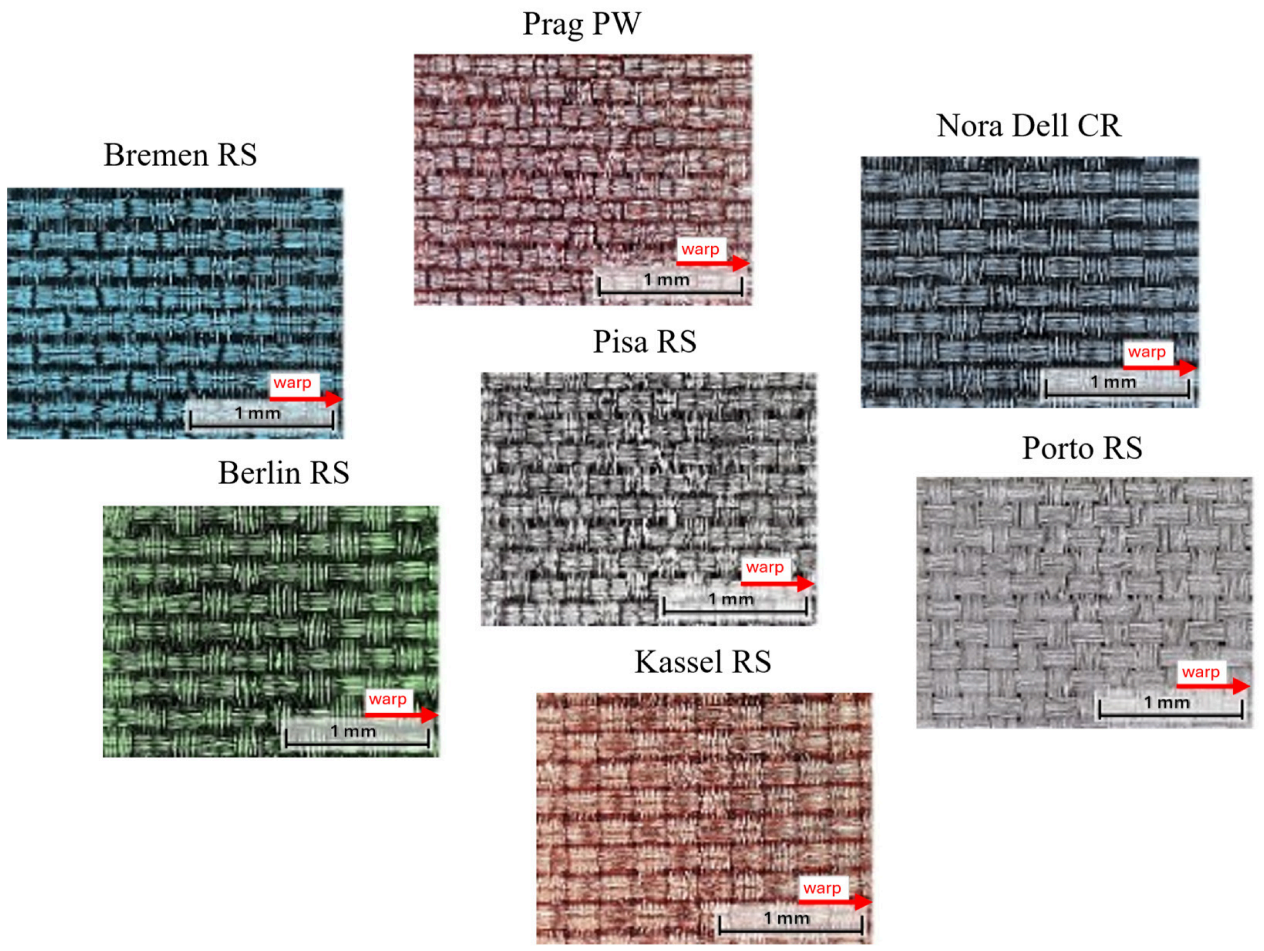
Optical microscopy images of metal-coated woven fabrics.

Images of the fabrics are shown in Figure 1. The optical microscope Delta Optical Smart 5MP PRO (Delta Optical, Warsaw, Poland) and the software Delta Optical Smart Analysis Pro 1.0.0 were used.

The structural parameters of woven fabrics were determined (Table 2). Measurements were repeated five times, and the mean value and coefficient of variation were calculated. Circular fabric samples with a diameter of 10.0 cm were prepared to measure electrical resistance in different directions within the sample plane.

## 3. Methods

### 3.1. Electrical Resistance

**Figure 2:**
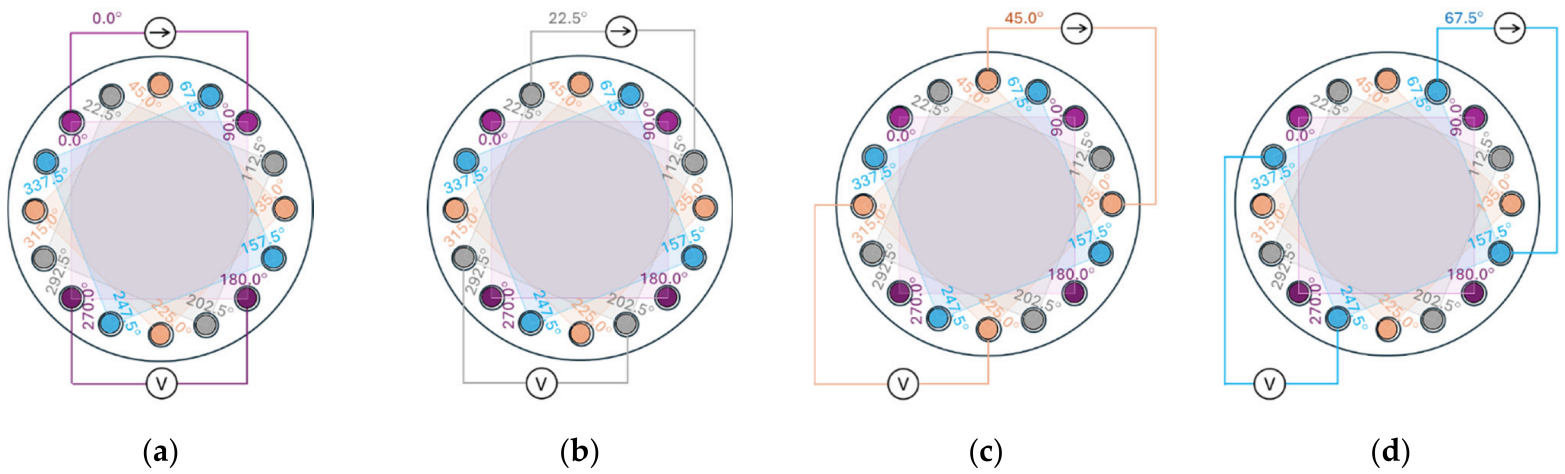
Idea of multidirectional resistance measurement: (**a**) Angle 0.0°—where the assumption was made that the line connecting the current electrodes is parallel to the weft direction; (**b**) Angle 22.5°; (**c**) Angle 45.0°; (**d**) Angle 67.5°.

The electrical resistance of the fabric samples was determined using a four-electrode configuration inspired by the van der Pauw method [50]. Four cylindrical brass electrodes were placed on the sample surface, forming a square with a side of 5.5 cm. The electrodes had a diameter of 2 mm to minimize the contact surface with the textile substrate. The force applied by a single electrode was 0.24 N, corresponding to a contact pressure of 75 kPa, which ensured stable electrode–substrate electrical contact. The starting point was assumed and designed as 0.0° (Figure 2a). Electric current was fed between two adjacent electrodes, and the voltage drop was measured between the other two. The resistance was calculated using Ohm’s law. Then, the electrode configuration was selected, corresponding to 22.5° (Figure 2b), and the electrical resistance was determined. The multidirectional resistance measurements were carried out in the range from 0 to 360°, with increments of 22.5°. An angular increment of 22.5° was selected to provide higher directional resolution and capture potential anisotropic effects more accurately. The electrode arrangements corresponding to the next two directions, i.e., 45.0° and 67.5°, are shown in Figure 2c and Figure 2d, respectively.

The fabric characteristics were determined based on multidirectional resistance measurements in the sample plane.

For the analysis of electrical properties, the relative resistance range was used as a measure of resistance variation and was calculated using the following formula:

(1) $$R_{N}=\frac{R_{max}-R_{min}}{R_{mean}},$$

where R_min_, R_max_, and R_mean_ are the minimum, maximum, and arithmetic mean of an electrical resistance, respectively, wherein Δ = R_max_ − R_min_ is a parameter that quantifies the electrical resistance anisotropy of an electroconductive woven fabric.

### 3.2. X-Ray Micro-Computed Tomography

X-ray micro-computed tomography (micro-CT, SkyScan 1272; Bruker, Kontich, Belgium) is a method based on the contrast of X-ray absorption that enables 3D characterization of materials. This technique can be applied to textile materials to determine structural parameters, such as weave type, warp and weft densities, and porosity, and it can also identify yarn cross-sections [51,52]. Micro-CT also enabled the identification of fabric cross-section shapes and their quantitative description. By revealing the internal structure, the technique enabled the identification of different materials within a sample.

The metal-coated woven fabrics were scanned under identical conditions, as shown in Table 3. A global pore and yarn analysis was performed on each fabric sample over an area of approximately 4 cm^2^ (2 cm × 2 cm). Since all sample cross-sections separated by a single voxel were included in the analysis, the dataset comprised 6666 cross-sections.

Micro-computed tomography was used for the visualization of the internal fabric structure and quantitative assessment of the fabric structure geometry. The fabric porosity P, metal coating thickness d, and statistical distributions of 3D-measured pore and yarn sizes were determined. For the yarn and pore size distribution analysis, the normalized span was used:

(2) $$S_{N}=\frac{D90-D10}{D50},$$

where D10, D50 (median), and D90 are the percentiles indicating that 10% of the yarn (or pore) sizes are smaller than D10, 50% are smaller than D50, and 90% are smaller than D90, respectively. A low normalized span value (S_N_ ≤ 1) indicates a narrow distribution of pore or yarn sizes, with values closely clustered around the median (D50). A high span value (S_N_ > 1) reflects a broad distribution of pore or yarn sizes, indicating substantial variability. A normalized span S_N_ = 0 theoretically corresponds to identical pore or yarn sizes across the entire dataset.

### 3.3. Scanning Electron Microscope-Energy-Dispersive X-Ray Spectroscopy

The fabric surface was studied using a scanning electron microscope-energy-dispersive X-ray spectroscopy (SEM-EDS) method. The microscope JEOL JSM-6610 LV (Jeol, Tokyo, Japan) was equipped with an X-MAX 80 module (Oxford Instruments, High Wycombe, UK). The module is a high-performance energy-dispersive X-ray spectrometer (EDS) designed for elemental analysis of materials. The analysis was carried out by applying an accelerating voltage of 20 kV and adjusting the beam current to obtain the sharpness of the analyzed image and a meaningful signal for chemical composition analysis. SEM-EDS analysis was used to confirm the chemical composition of the metal coatings deposited on the fabric surface, while SEM imaging was employed as a complementary technique to visualize the surface morphology of the fibers and yarns.

## 4. Results

### 4.1. Characterization of Metal Coating

SEM-EDS was used to identify the metal particles on the surface of the fabrics. The weight percentage (wt.%) of the chemical elements on the surface was determined, and the results are presented in Table 4.

The SEM-EDS elemental mapping images of the woven fabric yarn surface showing the distribution of the main chemical elements are presented in Table 5.

When comparing the data declared by the manufacturer with the results of the SEM-EDS analysis, some discrepancies were noticed. The EDS method, in essence, may show some errors when determining the shares of light and heavy elements simultaneously, which should be considered [53,54]. It applies especially to C, N, and O in combination with metals. The other problem is the contamination of samples, especially with carbon-based materials, which can contain O, N, and sometimes other compounds. The chamber itself will be contaminated with carbon material. Considering so many sources of additional elements and different sensitivities of their quantification, differences may result between declared data and analysis results. Even if the carbon C is cut off from the results, the rest from molecules and gases adsorbed on the surface, and products of chemical processes remain. This is quite natural and recognizable for the SEM-EDS method. The samples reflect a trace of the entire history of their processing and storage, and when directly comparing the results, such differences should be expected. The analysis showed metal coatings on the surface of all samples, ensuring their good electrical conductivity. The absence of Ag in the Kassel RS and Nora Dell CR samples may be due to its presence beneath other metal layers.

**Figure 3:**
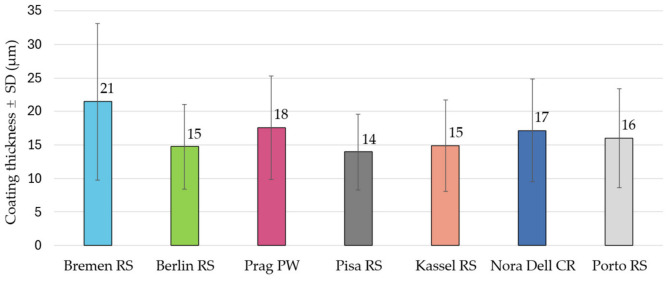
Mean coating thickness of woven fabrics.

Micro-CT was used to identify the thickness, d, of the electroconductive coating occurring on the fabric. The mean coating thickness d is shown in Figure 3. The standard deviation (SD) indicates the dispersion of coating thickness values around the mean, which ranges from 14 to 21 µm for all samples. The variation coefficient ranges from 41 to 54%, with the lowest being for the Pisa RS and the highest for the Bremen RS sample. This indicates that the metal coating on the fabric has an uneven thickness.

**Figure 4:**
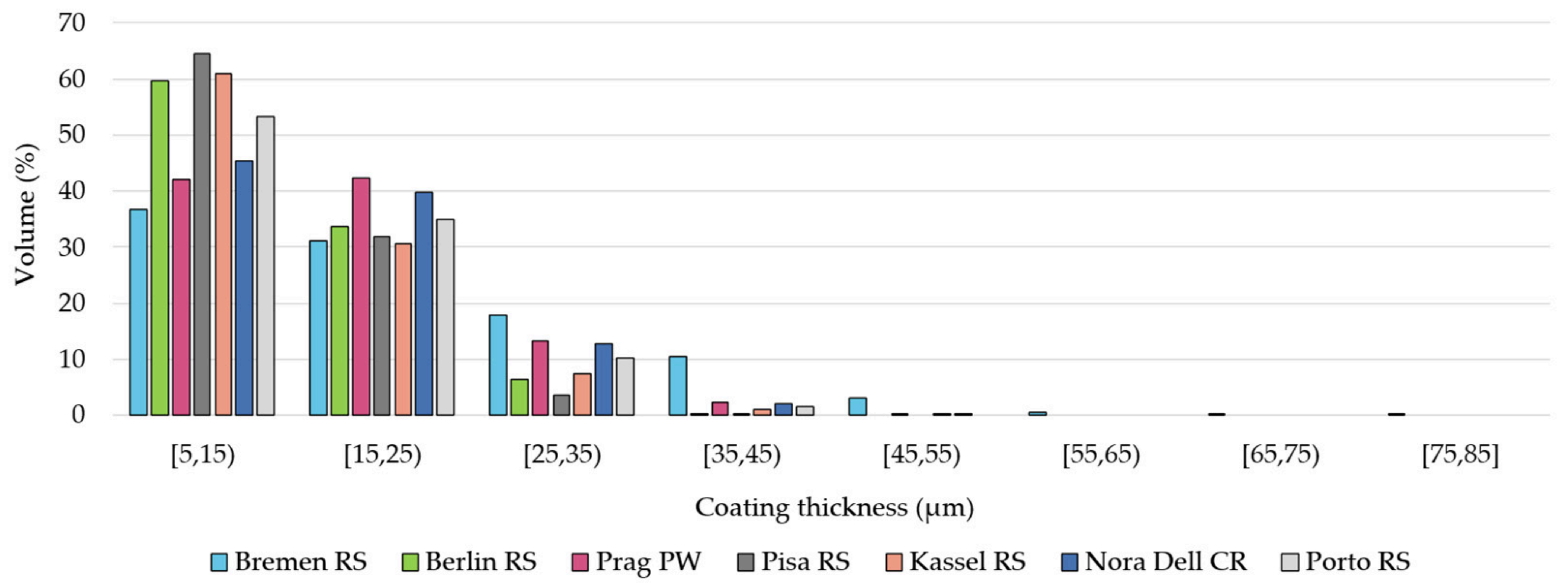
Distribution of coating thickness.

Next, the thicknesses were grouped into eight size classes to determine the thickness distribution. The thicknesses ranged from 5 to 85 µm. The results are presented in Figure 4.

The coating thickness d falls into the first two classes, with ranges of 5 to 15 µm and 15 to 25 µm. The two classes account for 68% of the total volume for the Bremen RS, 84–88% for the Prag PW, Nora Dell CR, and Porto RS, 91–93% for Kassel RS and Berlin RS, and 96% for Pisa RS. Therefore, the thickness of the metal coating on fabrics is mostly in the range of 5–25 µm. A voxel size of 3 µm and a measured minimum coating thickness of 5 µm (Figure 4) indicate that the metallic layer is clearly detectable throughout the fabric surface. Although this confirms the presence of the coating, a thickness measurement alone does not fully verify its microscale morphological continuity. Defects such as microcracks or very small discontinuities below the resolution limit may not be detected. Therefore, multidirectional resistance measurements were carried out as a subsequent step to coating evaluation.

### 4.2. Electrical Properties of Fabrics

**Figure 5:**
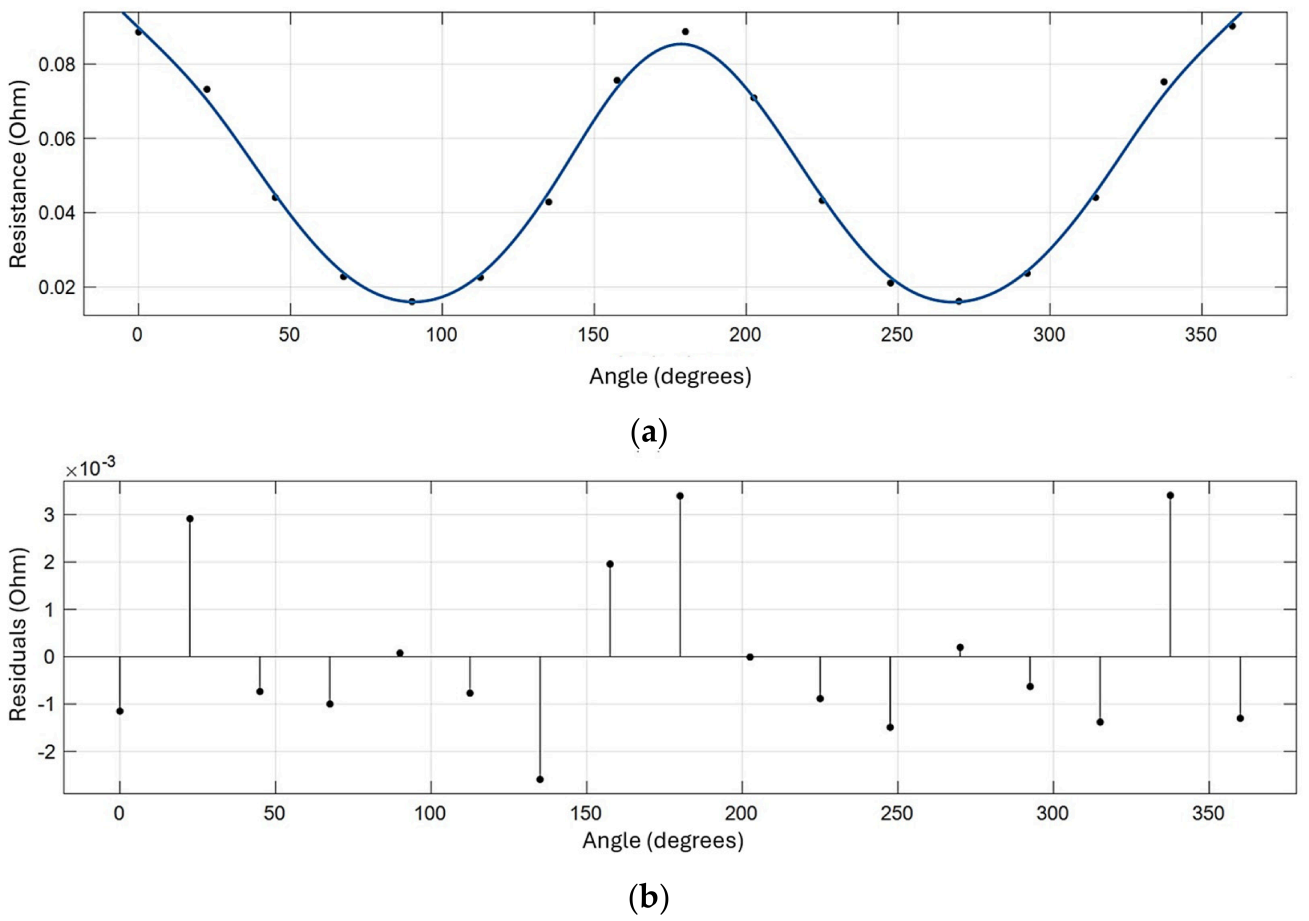
Bremen RS: (**a**) Anisotropy characteristic; (**b**) Residuals.

**Figure 6:**
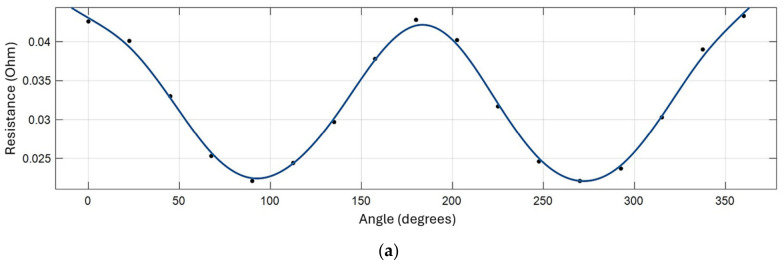

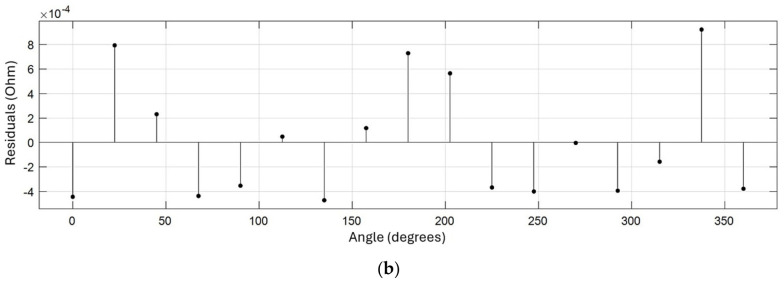
Berlin RS: (**a**) Anisotropy characteristic; (**b**) Residuals.

**Figure 7:**
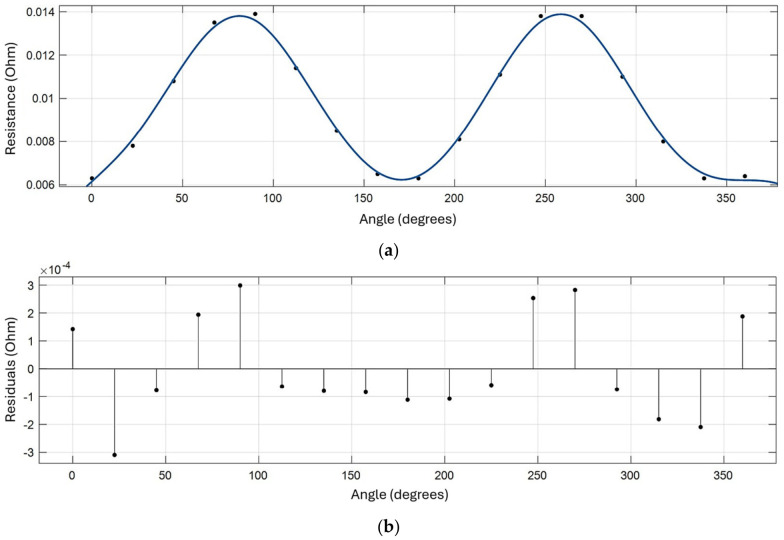
Prag PW: (**a**) Anisotropy characteristic; (**b**) Residuals.

**Figure 8:**
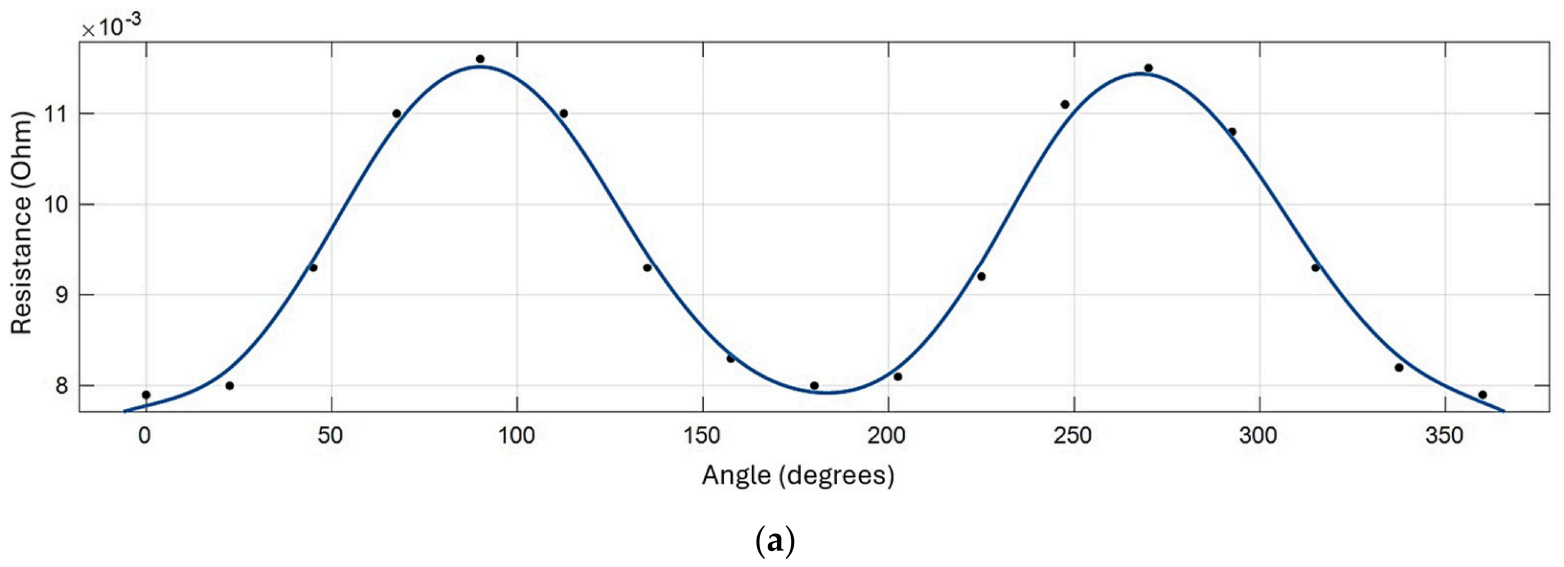

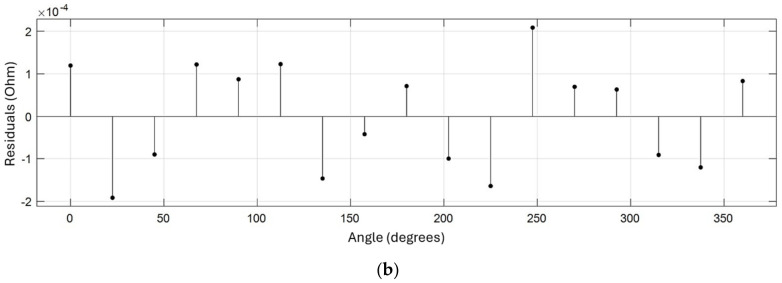
Pisa RS: (**a**) Anisotropy characteristic; (**b**) Residuals.

**Figure 9:**
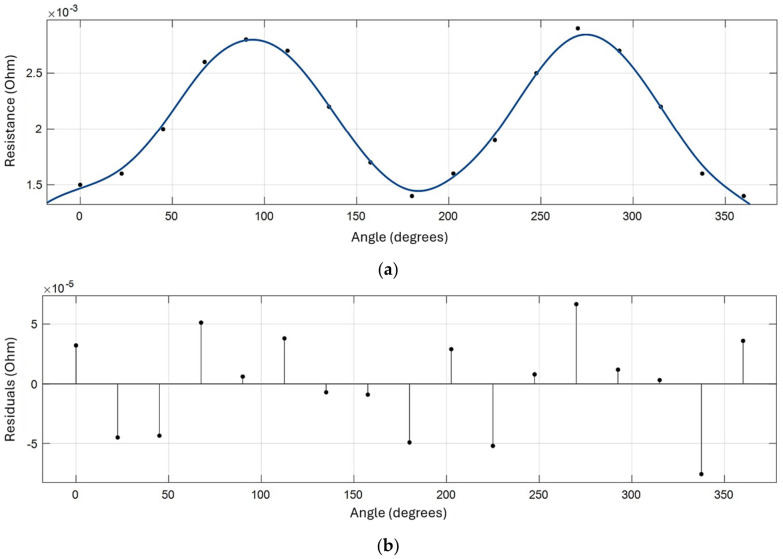
Kassel RS: (**a**) Anisotropy characteristic; (**b**) Residuals.

**Figure 10:**
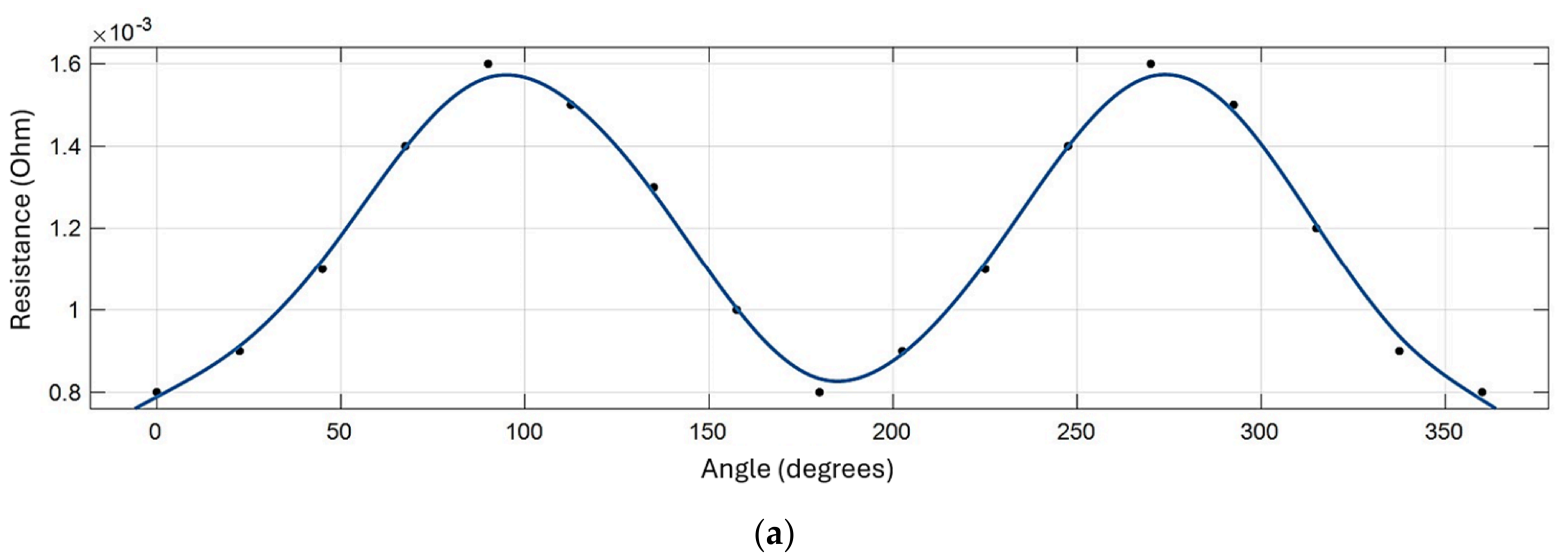

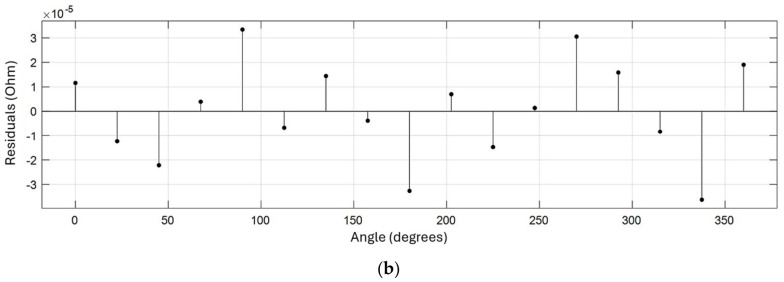
Nora Dell CR: (**a**) Anisotropy characteristic; (**b**) Residuals.

**Figure 11:**
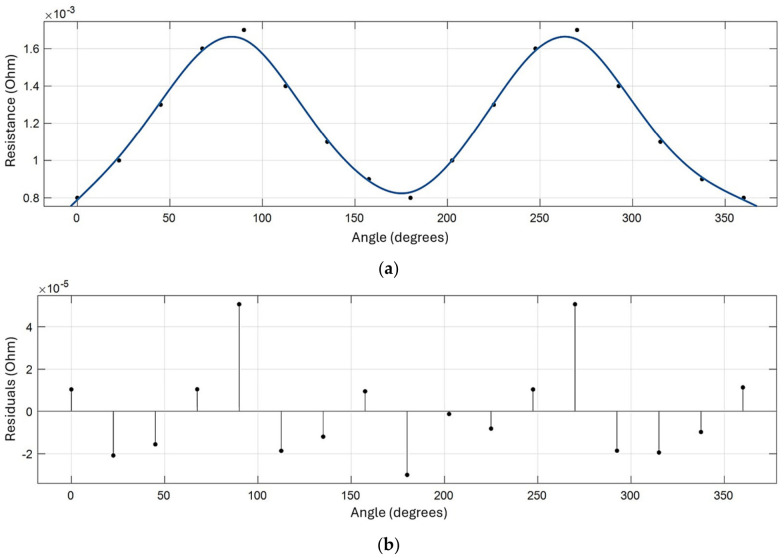
Porto RS: (**a**) Anisotropy characteristic; (**b**) Residuals.

Multidirectional resistance measurements were conducted for the metal-coated woven fabrics to determine whether direction-dependent electrical resistance exists. A temperature of 22.3 °C and relative air humidity of 55% were chosen as the testing conditions, as they are representative of typical indoor environmental conditions. The electrodes were arranged on the sample surface (Figure 2a) so that the lines connecting the current electrodes were perpendicular to the warp direction in the fabric. The measurements were repeated three times. The coefficient of variation did not exceed 6%. The anisotropy characteristics of the metal-coated fabrics are shown in Figure 5a, Figure 6a, Figure 7a, Figure 8a, Figure 9a, Figure 10a and Figure 11a. Smoothing spline regression was used in MATLAB (MathWorks, Natick, MA, USA, ver. R2021b) to fit the curves. Splines were used to approximate anisotropy curves because they provide a flexible representation of the measurement results. Experimental data slightly deviated from ideal periodicity, exhibiting variation in the widths and location of the maxima and minima. Even with careful electrode placement, small deviations from the intended electrode position on the sample surface cannot be eliminated, potentially affecting local electrical resistance measurements. Additionally, the residuals, which are the difference between the actual resistance value and the value predicted by the model for given points, are shown in Figure 5b, Figure 6b, Figure 7b, Figure 8b, Figure 9b, Figure 10b and Figure 11b.

Parameters determining the goodness of the curve fit, such as R-square—the coefficient of determination, SSE—the sum squared error, and RMSE—the root mean squared error, are shown in Table 6. The smoothing parameter for the fit type “smoothing spline” was equal to 0.0008 for all cases.

Cyclic changes in fabric resistance were observed in all metalized woven fabrics. In multidirectional electrical resistance measurements, the starting point (0 degrees) is associated with the electrodes’ arrangement. It was assumed that the line connecting the current electrodes is parallel to the sample weft direction. For all the tested woven fabrics, the warp density was greater than the weft density (Table 2). Meanwhile, one would expect the same extremum (minimum or maximum) to be achieved along the same yarn direction (either warp or weft) for all fabrics. However, the anisotropy curves indicate that the extrema are not always aligned with the same yarn direction (warp or weft). The maximum resistance values for 0 degrees were observed for Bremen RS (Figure 5a) and Berlin RS (Figure 6a). The minimum resistance values for 0 degrees were observed for Prag PW (Figure 7a), Pisa RS (Figure 8a), Kassel RS (Figure 9a), Nora Dell CR (Figure 10a), and Porto RS (Figure 11a). Thus, extreme values of electrical resistance correspond to the arrangement of the warp and weft threads, which are perpendicular to each other in the woven structure (Figure 1). The multidirectional resistance measurements were statistically analyzed in TIBCO Statistica 13.3 software to compare the electroconductive properties of fabrics. The Kruskal–Wallis test [55] was performed with a significance level of 0.05, and the *p*-value was used to estimate the probability of rejecting the null hypothesis (that the population medians are equal) when the hypothesis is true. A *p*-value of less than 0.05 was obtained, indicating significant differences in electrical conductivity between the woven fabrics. A post hoc test was used to identify pairs that differed significantly, assuming the same significance level. The results are presented in Table 7.

It was found that fabrics can be classified into three groups according to their electrical conductivity. There were no significant differences at the 0.05 significance level between all pairs in the group of the electroconductive woven fabrics:

I. Bremen RE, Berlin RS, Prag PW, and Pisa RS;
II. Prag PW, Pisa RS, and Kassel RS;
III. Kassel RS, Nora Dell CR, and Porto RS.

**Figure 12:**
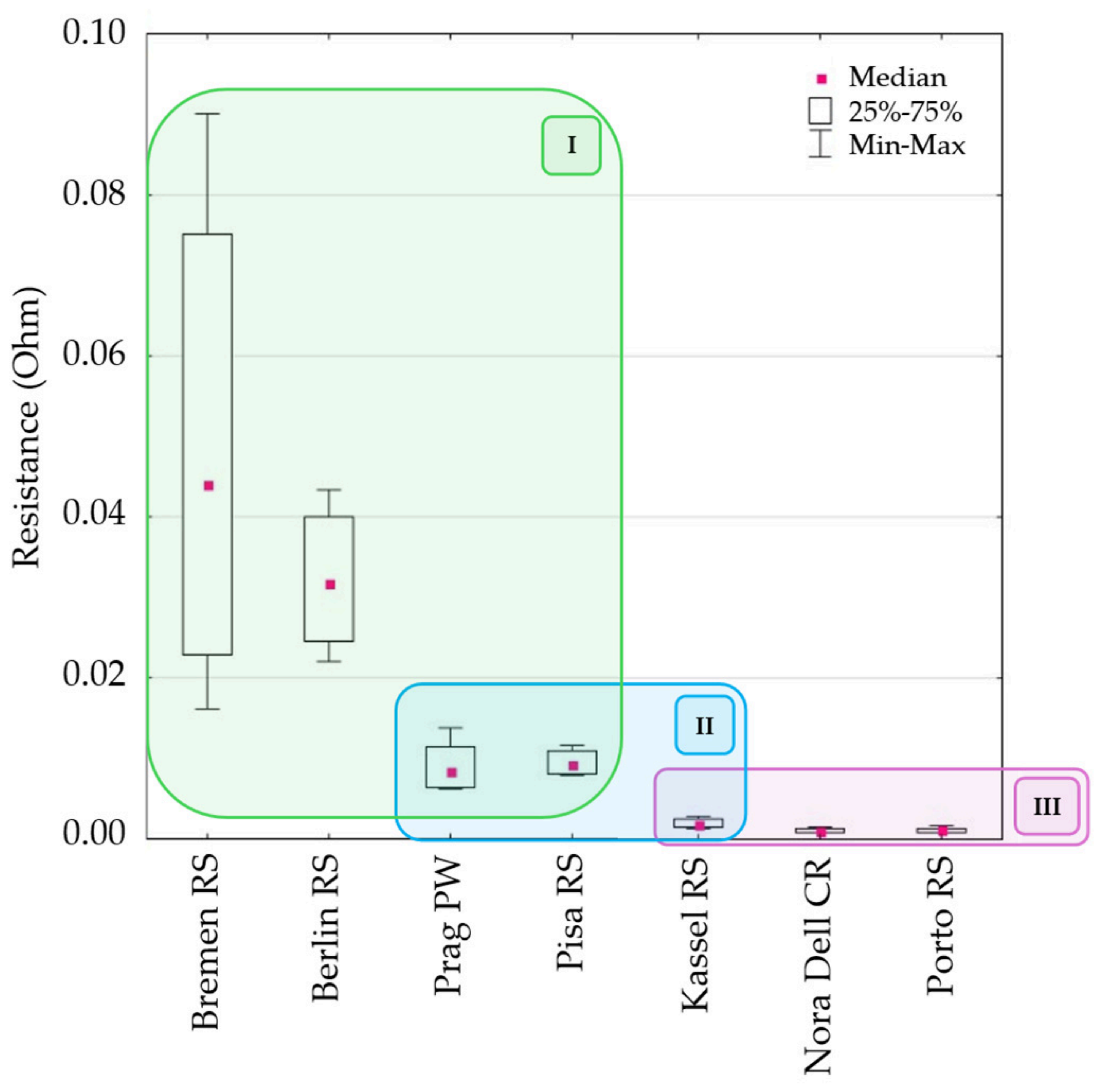
Median resistance values of electroconductive fabrics.

The results of the Kruskal–Wallis test are presented in graphical form (Figure 12), along with the division of fabrics into three groups (I, II, III; a different color indicates each group) based on their electroconductive properties.

**Figure 13:**
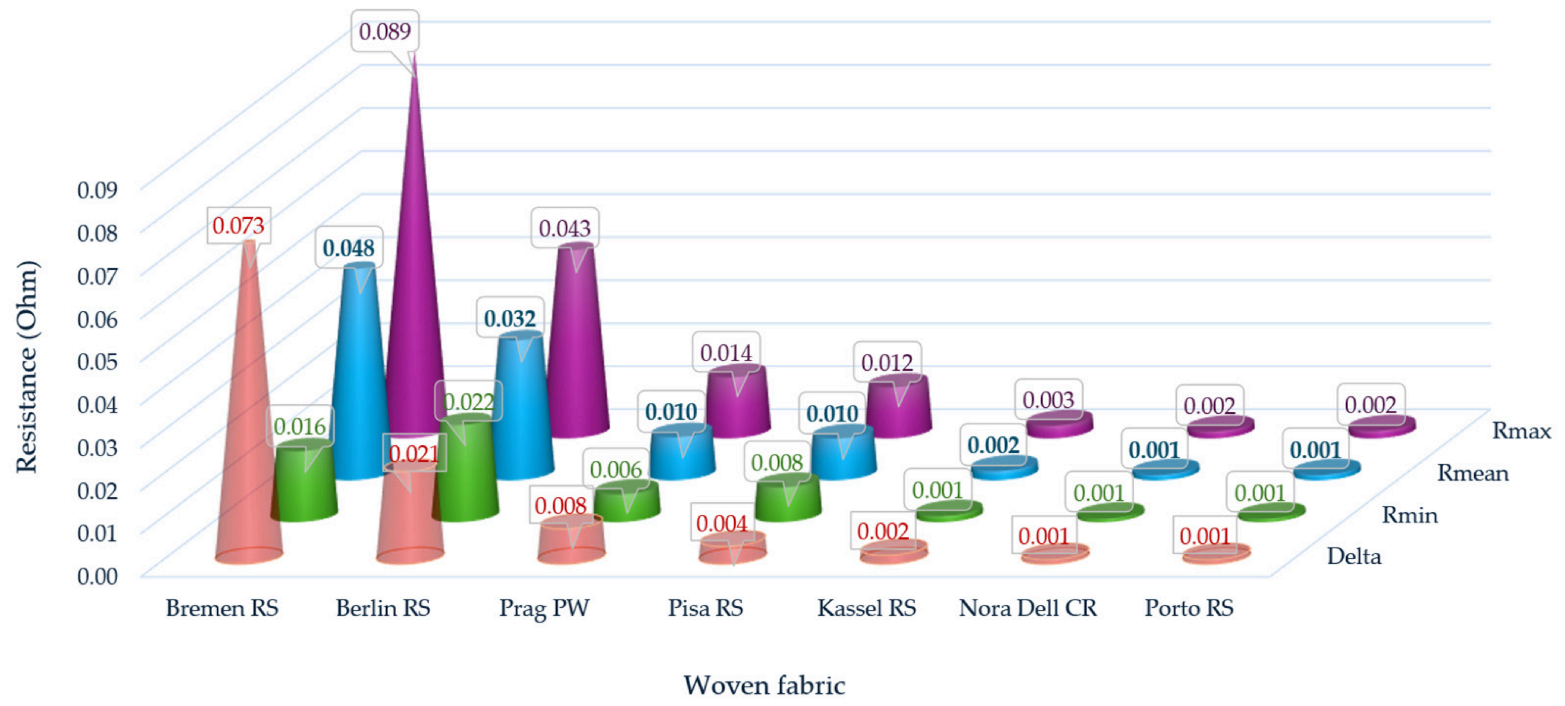
Electrical parameters of woven fabrics.

Parameters Δ, R_min_, R_max_, and R_mean_ were determined based on multidirectional fabric electrical resistance measurements and are shown in Figure 13. Fabrics are ranked from highest to lowest mean resistance; the term ‘Delta’ is used instead of the symbol Δ.

The multidirectional resistance measurements show that all investigated woven fabrics exhibited continuous electrical conduction in all measured directions. The highest electrical resistance value among all tested samples was 0.089 Ω, while the maximum difference between the highest and lowest directional resistance values was 0.048 Ω. These low resistance values indicate the presence of conductive pathways and suggest the absence of significant discontinuities in the conductive coating.

Woven fabrics such as Kassel RS, Nora Dell CR, and Porto RS (belonging to group III of fabrics, Figure 12), for which no statistically significant differences in median resistance were observed at a significance level of 0.05, exhibited the best electroconductive properties (Figure 13). The lowest Δ values were observed for these fabrics, indicating the lowest differences between the minimum and maximum in-plane resistivity values. Bremen RS and Berlin RS exhibited the highest mean resistance and the highest values of Δ. The relative resistance range parameter, R_N_ (Equation (1)), was additionally calculated. Results are presented in Table 8.

Porto RS is a fabric characterized by the lowest R_N_ value (0.4). The value may indicate more uniform electrical properties in the sample plane than in the remaining samples; nevertheless, its structural parameters are within the range of parameters of Prag PW, Pisa RS, Kassel RS, and Nora Dell CR fabrics. The R_N_ = 0.8 for Nora Dell CR, Pisa RS, and Prag PW demonstrates moderate variability in electrical resistance, reflecting differences in the electrical response depending on the measurement direction. Kassel RS, with an R_N_ value of 1.0, exhibits a similar level of resistance variability and can therefore be included in this group. It was found that Bremen RS and Berlin RS are characterized by the highest R_N_ values (1.5 and 1.4, respectively). A stronger directional dependence of electrical resistance compared to the previously discussed fabrics is observed. It should be emphasized that the Δ parameter provides information on the absolute difference between the maximum and minimum resistance values, whereas R_N_ enables a more reliable comparison of resistance variability between samples with different resistance values; however, both of them should be considered.

### 4.3. Micro-CT Characterization of Fabric Structure

**Figure 14:**
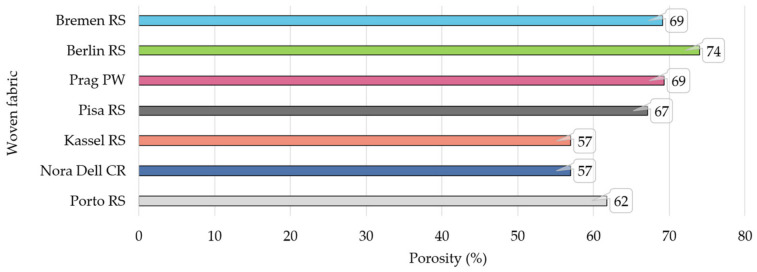
Woven fabrics’ porosity.

Characterization of the electroconductive woven fabrics using micro-CT was conducted. The porosity (P) of fabrics was determined and is shown in Figure 14.

The fabrics’ porosity values ranged from 57 to 74%. Despite their dense structure, the fabrics remain lightweight due to the significant contribution of void volume. To better understand the origin of this phenomenon, cross-sectional analysis was conducted along the warp and weft directions. The results are presented in Table 9. The cross-sectional shape of the warp yarns was identified as elliptical (Table 9, column ‘Cross-section view of the warp yarns’). The cross-sectional shape of the weft yarns was determined to be rectangular (Table 9, column ‘Cross-section view of the weft yarns’). The cross-sectional images reveal the presence of internal void spaces within the woven structure, which contribute to the high porosity of the fabrics.

The lengths of the minor and major axes of the ellipse and sides of the rectangle were determined and are shown in Table 10.

The average width of yarn in fabrics ranges from 135 to 180 µm for the warp (Table 10, column ‘Major axis’) and from 180 to 255 µm for the weft (Table 10, column ‘Longer side’). The major axis and the longer side lengths are directly related to yarn width.

**Figure 15:**
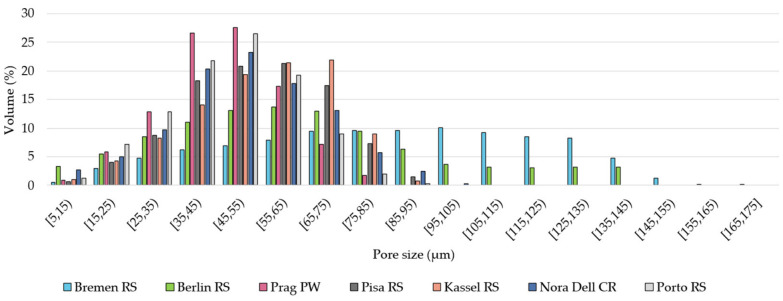
Distribution of pore sizes.

The pore sizes were identified and then grouped into 17 size classes ranging from 5 to 175 μm. Pore size distributions (PSD) in electroconductive fabrics are shown in Figure 15. Many pores of varying sizes were observed in Bremen RS and Berlin RS fabrics (Figure 15). Hence, these observations suggest a more heterogeneous woven structure. The distributions of the remaining fabrics are skewed towards the smaller pore size.

**Figure 16:**
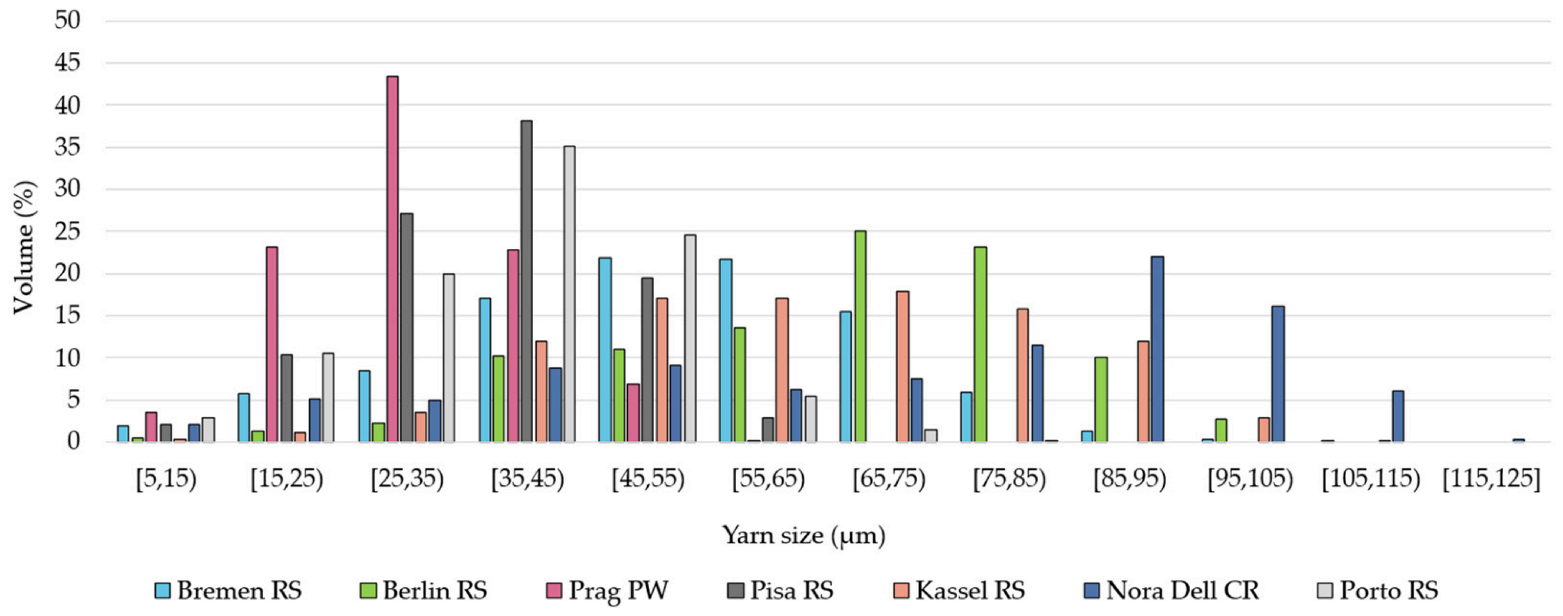
Distribution of yarn sizes.

A yarn size distribution (YSD) in fabrics was also determined. The distributions are shown in Figure 16, where 12 size classes were identified in the range of 5 to 125 μm. The yarn size distributions reveal a relatively broad range of yarn sizes of all woven fabrics (Figure 16), indicating heterogeneity of the fabric structure.

The plot in Figure 16 represents the yarn thickness distribution, not the fiber thickness. The fine, compact yarn structure makes it impossible to identify and calculate the contribution of individual fiber thickness to the total yarn thickness. Fibers are densely packed within the thin yarn, as confirmed by SEM images (Table 5), and can form yarn with a very flattened cross-section perpendicular to the fabric surface, as confirmed by micro-CT images (Table 9). Fiber identification is severely limited by the presence of a metallic coating on the fabric, which contributes to X-ray blurring of the yarn surface, even when scanning samples at high resolution (Table 9).

The observed variability in pore and yarn sizes needs quantitative assessment using dedicated metrics. Based on micro-CT analysis of pores and yarns in woven fabrics, characteristic percentiles, arithmetic mean yarn/pore size, and normalized span (Equation (2)) were determined. The results are presented in Table 11, where metrics relevant for further analysis of yarn and pore sizes were identified: D50Y—the median yarn size, YS—the yarn size, S_N_Y—the normalized span of the yarn size distribution, D50P—the median pore size, PS—the pore size, and S_N_P—the normalized span of the pore size distribution. The coefficient of variation of yarn size (YS) ranged from 27 to 38%, whereas that of pore size (PS) ranged from 30 to 50%.

It was observed that for two woven fabrics, the normalized span calculated for pores is the highest and exceeds the value 1, with S_N_P = 1.2 for Bremen RS and S_N_P = 1.5 for Berlin RS (Table 11), indicating high variability in pore sizes. The fabrics are characterized by the highest minimum and maximum electrical resistances, R_min_ = 0.016 Ω, R_max_ = 0.089 Ω, and R_min_ = 0.022 Ω, R_max_ = 0.043 Ω (Bremen RS and Berlin RS samples, respectively, see Figure 13), as well as the highest porosity, 69% and 74% for Bremen RS and Berlin RS, respectively (Figure 14). A narrower distribution of pore sizes (S_N_P ranged from 0.7 to 0.9), with sizes closely clustered around the median (Figure 15), and lower electrical resistance values (Figure 13) were observed for the remaining samples. However, in all investigated samples, it was noticed that the yarn sizes are clustered around the median (S_N_Y ranged from 0.8 to 0.9).

## 5. Discussion

Correlation analysis was conducted using coating thickness, selected PSD and YSD parameters, structural parameters, and electrical parameters. The results of the analysis are juxtaposed in Table 12. 

**Figure 17:**
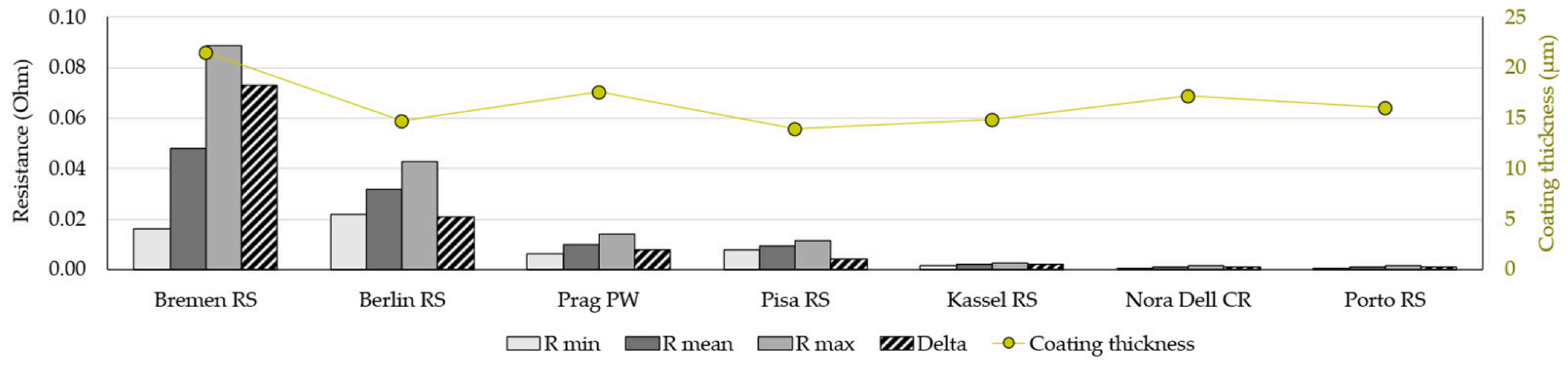
Resistances vs. metal coating thickness. Line guides the eye.

A strong positive correlation is observed between metal coating thickness d and electrical parameter Δ (R_p_ = 0.772). This suggests that woven fabrics with thicker coatings tend to be associated with greater differences between the minimum and maximum in-plane resistivity values. This may indicate that the deposited metal preferentially accumulates on the raised regions of the fabric surface, resulting from fiber/yarn interlacing, rather than being uniformly distributed over the entire fabric surface. The non-uniform coating distribution may contribute to increased anisotropy of the electrical properties. The lower correlation coefficient observed for coating thickness compared with the other parameters (Table 12) suggests that coating thickness may play a secondary role relative to woven fabric architecture in determining electrical properties. Figure 17 shows the Δ (Delta), minimum, median, and maximum electrical resistances, as well as the coating thickness for all the metalized woven fabrics.

**Figure 18:**
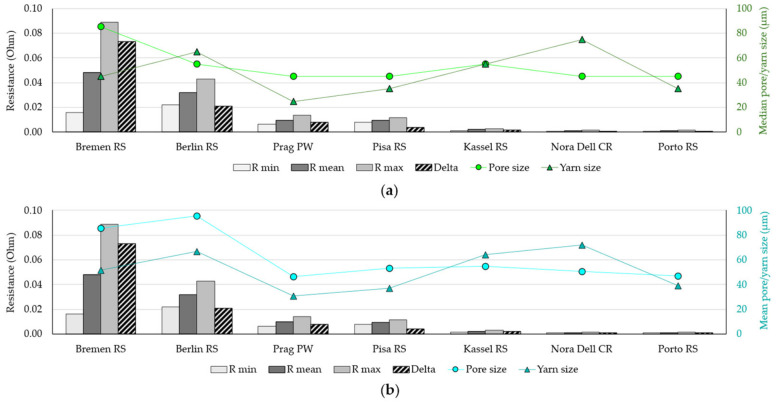
Resistances vs. pore/yarn size metrics: (**a**) Median size; (**b**) Mean size. Lines guide the eye.

There is a very strong positive correlation between median pore size D50P, mean pore size PS, and minimum resistance R_min_ (R_p_ = 0.839 and R_p_ = 0.907, respectively). Moreover, both D50P and PS exhibited strong positive correlations with the mean resistance R_mean_ (R_p_ = 0.856 and R_p_ = 0.882, respectively). Additionally, the correlation between PS and R_max_ is also very strong (R_p_ = 0.941). Taken together, these results suggest that the increase in pore size is associated with an increase in minimum and mean resistance (Figure 18). This may be related to the fact that an increase in pore sizes can be associated with a more heterogeneous pore structure, which can lead to a less uniform distribution of conductive pathways within the fabric, and consequently higher resistance values. In metal-coated woven fabrics, the conductive pathways consist of interconnected regions of the conductive coating on fiber/yarn surfaces and at fiber/yarn crossover points, which can form a continuous network for electrical current flow through the fabric.

**Figure 19:**
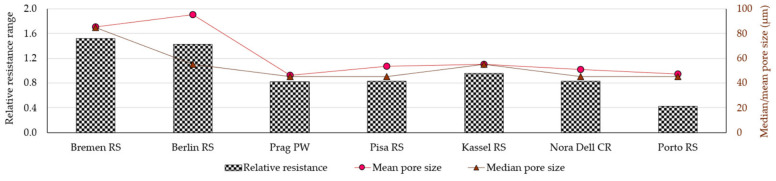
Relative resistance range parameter vs. median/mean pore size. Lines guide the eye.

It was found that a very strong positive correlation exists between the median pore size D50P and the parameter Δ (R_p_ = 0.958) (Figure 18a). Additionally, the median pore size D50P exhibited a strong positive correlation with the relative resistance range parameter R_N_ (R_p_ = 0.782), while the mean pore size PS exhibited a very strong positive correlation with R_N_ (R_p_ = 0.911) (Figure 19). Together with the very strong correlation observed between D50P and Δ (R_p_ = 0.958), these results suggest that an increase in pore sizes is associated with increased direction-dependent variability in the electrical response, reflecting an increase in electrical anisotropy in the sample plane.

**Figure 20:**
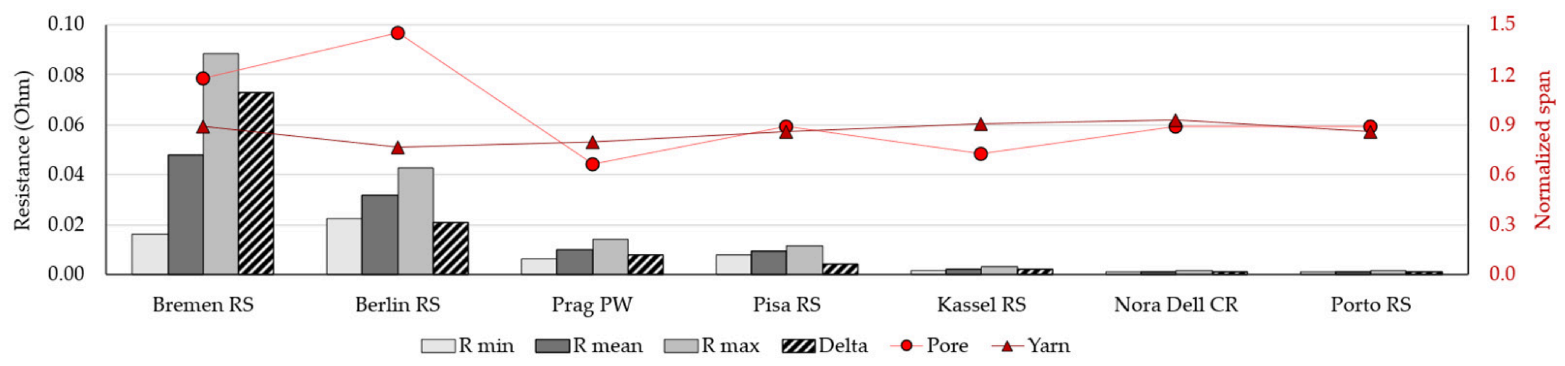
Resistances vs. normalized span. Lines guide the eye.

A strong positive relationship was identified between normalized span of the pore size distribution S_N_P and both minimum resistance R_min_ and maximum resistance R_max_ (R_p_ = 0.830 and R_p_ = 0.953, respectively) (Figure 20). A broader pore size distribution, reflecting greater variability in pore sizes, may be related to increased heterogeneity in the woven structure. This may contribute to higher minimum and maximum electrical resistance values.

**Figure 21:**
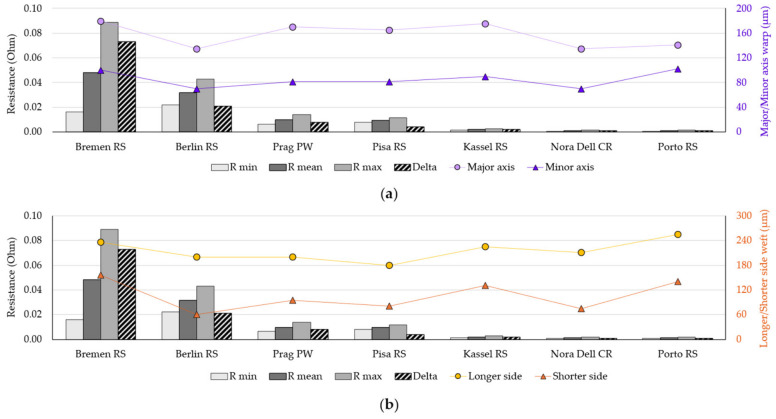
Resistances vs. yarn cross-section metrics: (**a**) Minor/major axis of warp; (**b**) Longer/shorter side of weft. Lines guide the eye.

No correlation was found between yarn size metrics (D50Y, YS, S_N_Y) and electrical parameters (Δ, R_min_, R_max_, R_mean_, R_N_). The comparison of pore and yarn metrics is presented in Figure 18 and Figure 20. No correlation was also found between yarn cross-section metrics (A_ma/wa_, A_mi/wa_, L_we_, S_we_), chosen fabric parameters (D_wa_, D_we_, P), and the electrical parameters. Figure 21 shows electrical resistances, as well as yarn cross-section metrics for all the investigated woven fabrics.

**Figure 22:**
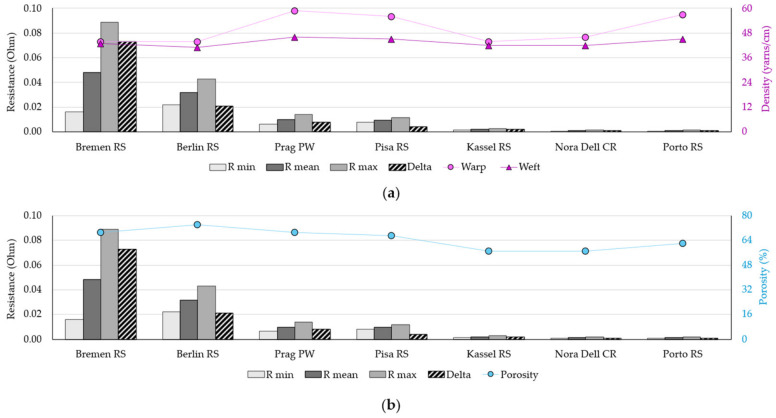
Resistances vs. yarn cross-section metrics: (**a**) Warp and weft densities; (**b**) Porosity. Lines guide the eye.

Figure 22 presents the electrical resistances, along with the selected structural parameters, for all investigated woven fabrics.

The investigated textile materials differed simultaneously in numerous structural parameters (e.g., fabric thickness, yarn cross-sectional shape and size, and the number of fibers in the yarn), as well as in raw material composition of yarn and coating parameters (e.g., coating composition and thickness). Therefore, this may explain the lack of correlation between selected yarn metrics determined by micro-CT analysis and the electrical properties of the woven fabrics.

The woven fabrics are made of PA and PES, onto which a metal layer has been deposited. Increased relative humidity may reduce electrical resistance due to enhanced moisture absorption of the sample, while temperature changes can alter charge transport within the metalized structure. Changes in relative humidity may affect the electrical resistance, particularly if the coating contains discontinuities or exposed polymer regions (e.g., along the cut edges, where the polymer substrate is exposed). PES (polyester) exhibits negligible moisture absorption, whereas PA (polyamide) is relatively hygroscopic. Temperature variations may influence the electrical resistance of woven fabrics, mainly through changes in electron transport within the coating. Increasing temperature may increase resistance due to reduced electron mobility in the metallic coating.

Investigating the electrical anisotropy of woven fabrics is important from the point of view of selecting materials for specified applications. Electrical isotropy is required for applications such as textile electrodes and electromagnetic shieldings, where the textile material should be characterized by very good electrical properties and uniform current distribution. It can be implemented in wearable e-textile systems, where electrically isotropic fabrics can be integrated without the need to control their orientation, allowing their placement to be optimized according to other design requirements. Anisotropy is advantageous in the case of heating elements and resistive sensors. It enables the choice of the appropriate resistance value. Higher electrical resistances are required for heating elements. However, the resistance cannot be too low, as it may result in excessive current, leading to overheating. The higher-resistance direction can be exploited to improve the sensitivity of resistive sensors.

## 6. Conclusions

The study demonstrates the potential of micro-CT for the comprehensive characterization of metal-coated woven fabrics. The following conclusions can be drawn from the research.

- Micro-CT analysis confirmed the presence of the metal coating on fabric surfaces. The coating thicknesses ranged from 14 to 21 μm, with a variation coefficient up to 54%. At the same time, multidirectional resistance measurements confirmed continuous electrical conduction and low electrical resistance values for all investigated woven fabrics; among all the fabrics, the maximum values of the parameters were R_max_ = 0.089 Ω and Δ = 0.048 Ω. This indicates the presence of conductive pathways and suggests no significant discontinuities in the conductive coating. Correlation analysis revealed that a thicker metal coating on the fabric surface may be associated with increased anisotropy of the electrical properties (parameter Δ), with a significant correlation coefficient of 0.772 at the 0.05 significance level.
- A very strong relationship was observed between the pore size characteristics determined by micro-CT analysis and the electrical parameters of metal-coated woven fabrics. The results suggest that an increase in pore sizes (mean and median) may be associated with higher resistance values (correlation coefficients ranged from 0.839 to 0.941 at the 0.05 significance level), potentially contributing to changes in the distribution of conductive regions within the fabric. An increase in pore sizes (median) may be associated with greater dimensional irregularity, which in turn may be related to increased direction-dependent variability in the electrical response, reflecting higher electrical anisotropy within the sample plane (correlation coefficient of 0.958 at the 0.05 significance level). Electrical resistance reached extreme values along the warp and weft directions.
- The two most porous metal-coated woven fabrics, Bremen RS and Berlin RS, with porosities of 69% and 74%, respectively, and highly variable pore sizes of 1.2 and 1.5, respectively, were characterized by extreme electrical resistance values.

## Figures and Tables

**Table 1:** Characteristics of electroconductive woven fabrics.

Fabric	Raw Material Composition	Surface Resistance (Ω/sq.)
Bremen RS	82% PA + 18% Ag	<0.30
Berlin RS	60% PA + 14% Ag + 26% PU	<0.05
Prag PW	46% PES + 49% Cu + 5% CR	≤0.05
Pisa RS	35% PES + 61% Cu + 4% Ni	≤0.05
Kassel RS	57% PA + 9% Ag + 49% Cu + 5% CR	≤0.03
Nora Dell CR	32% PA + 7% Ag + 48% Cu + 4% Ni + 9% CR	<0.01
Porto RS	31% PES + 65% Cu + 4% Sn	≤0.02

**Table 2:** Structural parameters of fabrics.

Fabric	Weave	Thickness h (mm)	Surface Mass SM (g/m^2^)	Warp Density D_wa_ (Yarns/cm)	Weft Density D_we_ (Yarns/cm)
Bremen RS	Ripstop	0.088 (1.0%)	40 (7%)	44	43
Berlin RS	Ripstop	0.100 (0.9%)	57 (6%)	44	41
Prag PW	Plain	0.075 (0.7%)	70 (5%)	59	46
Pisa RS	Ripstop	0.081 (1.1%)	75 (6%)	56	45
Kassel RS	Ripstop	0.090 (1.0%)	85 (5%)	44	42
Nora Dell CR	Ripstop	0.113 (1.0%)	119 (2%)	46	42
Porto RS	Ripstop	0.092 (1.0%)	122 (3%)	57	45

**Table 3:** High-resolution scanning conditions for the tested fabrics.

Parameter	Value
X-ray source voltage (kV)	90
X-ray source current (µA)	111
Image resolution (pixels)	4032 × 2688
Voxel size (µm)	3
Rotation step (°)	0.1
Averaging (number of images for a given angle)	5
Filter	Al 0.5 + Cu 0.038

**Table 4:** Fabric surface chemical composition expressed in wt.% (±SD ^1^).

Fabric	Ag	Cu	Ni	Sn	P	C	O
Bremen RS	27.3 ± 0.1	-	-	-	-	63.2 ± 0.1	9.5 ± 0.1
Berlin RS	38.4 ± 0.1	-	-	-	-	53.5 ± 0.1	8.1 ± 0.1
Prag PW	-	65.5 ± 0.1	-	-	-	31.1 ± 0.1	3.4 ± 0.1
Pisa RS	-	68.1 ± 0.1	14.9 ± 0.1	-	-	17.0 ± 0.1	-
Kassel RS	-	56.9 ± 0.1	-	-	-	38.4 ± 0.1	4.7 ± 0.1
Nora Dell CR	-	35.8 ± 0.1	32.3 ± 0.1	-	1.7 ± 0.1	27.6 ± 0.1	2.6 ± 0.1
Porto RS	-	42.8 ± 0.1	-	43.8 ± 0.1	-	10.4 ± 0.1	3.0 ± 0.1

^1^ The standard deviation.

**Table 5:** SEM surface images and SEM-EDS elemental mapping images of electroconductive woven fabrics.

Fabric	SEM Image/Color Map of Main Element Distribution
Bremen RS	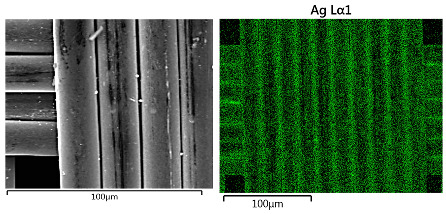
Berlin RS	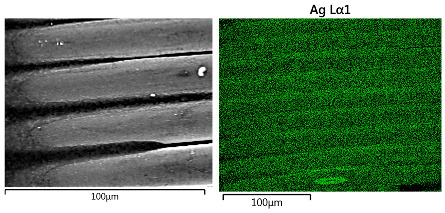
Prag PW	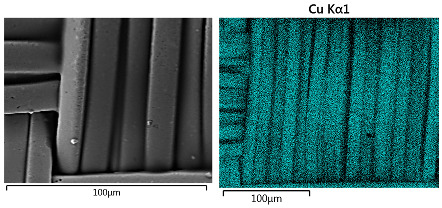
Pisa RS	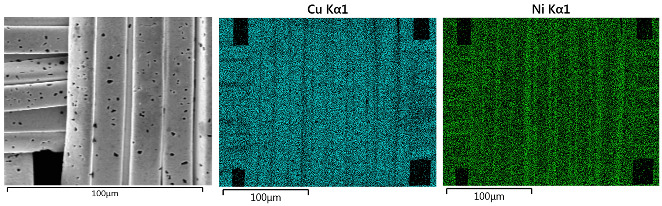
Kassel RS	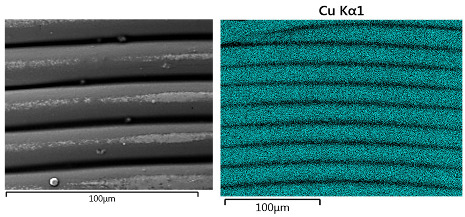
Nora Dell CR	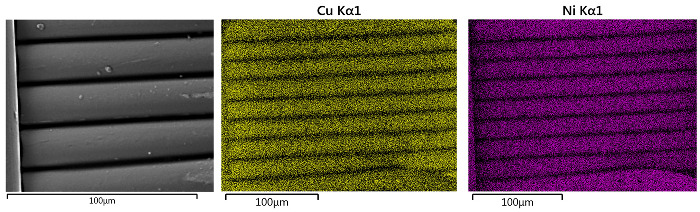
Porto RS	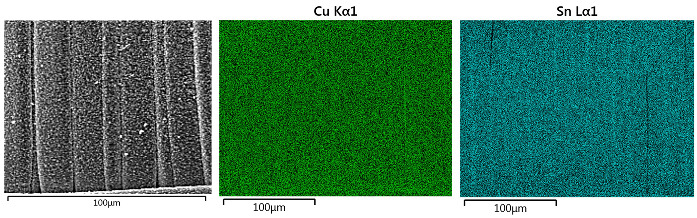

**Table 6:** Curve fit quality parameters.

Fabric	R-Square	SSE (Ω^2^)	RMSE (Ω^2^)
Bremen RS	0.9957	5.269 × 10^−5^	2.930 × 10^−3^
Berlin RS	0.9962	3.756 × 10^−6^	7.821 × 10^−4^
Prag PW	0.9960	5.553 × 10^−7^	3.007 × 10^−4^
Pisa RS	0.9924	2.439 × 10^−7^	1.993 × 10^−4^
Kassel RS	0.9941	2.681 × 10^−8^	6.608 × 10^−5^
Nora Dell CR	0.9953	6.441 × 10^−9^	3.239 × 10^−5^
Porto RS	0.9949	8.622 × 10^−9^	3.747 × 10^−5^

**Table 7:** The *p*-values as results of the post hoc test ^2^.

Fabric	Bremen RS	Berlin RS	Prag PW	Pisa RS	Kassel RS	Nora Dell CR	Porto RS
Bremen RS		1.000	0.167	0.203	**0.000**	**0.000**	**0.000**
Berlin RS	1.000		0.531	0.632	**0.000**	**0.000**	**0.000**
Prag PW	0.167	0.531		1.000	0.164	**0.000**	**0.000**
Pisa RS	0.203	0.632	1.000		0.134	**0.000**	**0.000**
Kassel RS	**0.000**	**0.000**	0.164	0.134		0.879	1.000
Nora Dell CR	**0.000**	**0.000**	**0.000**	**0.000**	0.879		1.000
Porto RS	**0.000**	**0.000**	**0.000**	**0.000**	1.000	1.000	

^2^ The *p*-values marked in bold indicate significantly differing pairs of fabrics.

**Table 8:** Relative resistance range parameter.

Fabric	Bremen RS	Berlin RS	Prag PW	Pisa RS	Kassel RS	Nora Dell CR	Porto RS
R_N_	1.5	1.4	0.8	0.8	1.0	0.8	0.4

**Table 9:** Cross-section of fabrics.

Fabric	Cross-Sectional View of Warp Yarns	Cross-Sectional View of Weft Yarns
Bremen RS	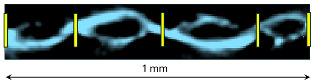	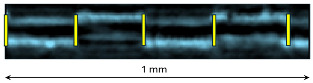
Berlin RS	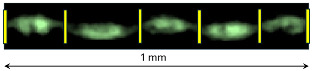	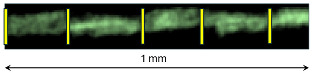
Prag PW	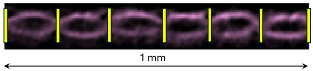	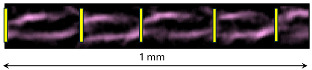
Pisa RS	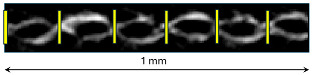	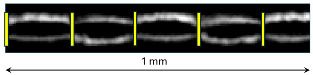
Kassel RS	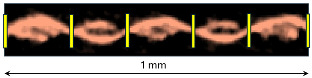	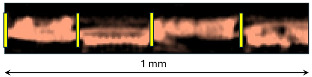
Nora Dell CR	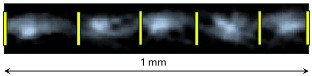	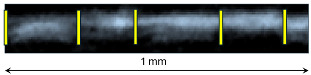
Porto RS	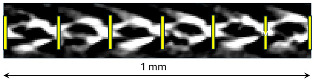	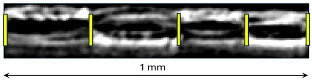

**Table 10:** Quantitative description of cross-section of yarns.

Fabric	Warp		Weft	
	Major Axis A_ma/wa_ (µm)	Minor Axis A_mi/wa_ (µm)	Longer Side L_we_ (µm)	Shorter Side S_we_ (µm)
Bremen RS	180	100	235	155
Berlin RS	135	70	200	60
Prag PW	170	81	200	95
Pisa RS	165	82	180	80
Kassel RS	175	90	225	130
Nora Dell CR	135	70	210	75
Porto RS	141	102	255	140

**Table 11:** Pore and yarn size metrics.

Fabric	Yarn						Pore					
	D10 (µm)	D50Y (µm)	D90 (µm)	D90-D10 (µm)	YS (µm)	S_N_Y	D10 (µm)	D50P (µm)	D90 (µm)	D90-D10 (µm)	PS (µm)	S_N_P
Bremen RS	25	45	65	40	52	0.9	35	85	135	100	85	1.2
Berlin RS	35	65	85	50	67	0.8	25	55	105	80	95	1.5
Prag PW	15	25	35	20	31	0.8	25	45	55	30	46	0.7
Pisa RS	15	35	45	30	37	0.9	25	45	65	40	53	0.9
Kassel RS	35	55	85	50	64	0.9	25	55	65	40	55	0.7
Nora Dell CR	25	75	95	70	72	0.9	25	45	65	40	51	0.9
Porto RS	15	35	45	30	39	0.9	25	45	65	40	47	0.9

**Table 12:** Values of Pearson’s correlation coefficients (R_p_) ^3^.

Parameters Structure → Electrical ↓	D50Y	S_N_Y	D50P	S_N_P	YS	PS	P	d	A_ma/wa_	A_mi/wa_	L_we_	S_we_	D_wa_	D_we_
Δ	−0.019	−0.004	**0.958**	0.545	0.054	0.708	0.482	**0.772**	0.427	0.366	0.237	0.449	−0.436	−0.194
R_min_	0.356	−0.118	**0.839**	**0.830**	0.391	**0.907**	0.529	0.581	0.031	0.022	0.130	0.118	−0.653	−0.531
R_max_	0.463	−0.505	0.443	**0.953**	0.468	**0.941**	0.661	0.107	−0.340	−0.322	−0.089	−0.297	−0.575	−0.638
R_mean_	0.042	−0.298	**0.856**	0.751	0.095	**0.882**	0.722	0.570	0.270	0.128	0.013	0.167	−0.446	−0.287
R_N_	0.359	−0.191	**0.782**	0.726	0.431	**0.911**	0.558	0.391	0.242	−0.168	−0.179	−0.046	−0.718	−0.597

^3^ Significant correlation coefficients at a 0.05 significance level are marked in bold.

## Data Availability

The original contributions presented in this study are included in the article. Further inquiries can be directed to the corresponding author.

## References

[1] Castano L.M., Flatau A.B. (2014). Smart fabric sensors and e-textile technologies: A review. Smart Mater. Struct..

[2] Sindhu G., Poornima A. (2024). A brief synopsis on conductive textiles. Int. J. Adv. Biochem. Res..

[3] Agcayazi T., Chatterjee K., Bozkurt A., Ghosh T.K. (2018). Flexible interconnects for electronic textiles. Adv. Mater. Technol..

[4] Leśnikowski J. (2024). Influence of repeated switching of current through contacts made of electroconductive fabrics on their resistance. Autex Res. J..

[5] Stanley J., Hunt J.A., Kunovski P., Wei Y. (2022). A review of connectors and joining technologies for electronic textiles. Eng. Rep..

[6] Petru M., Ali A., Khan A.S., Srb P., Kucera L., Militky J. (2023). Flexible coated conductive textiles as ohmic heaters in car seats. Appl. Sci..

[7] Havelka A., Nagy L., Tunák M., Antoch J. (2021). Testing the effect of textile materials on car seat comfort in real traffic. J. Ind. Text..

[8] Atalay O., Kalaoglu F., Kursun Bahadir S. (2019). Development of textile-based transmission lines using conductive yarns and ultrasonic welding technology for e-textile applications. J. Eng. Fiber. Fabr..

[9] Du K., Lin R., Yin L., Ho J.S., Wang J., Lim C.T. (2022). Electronic textiles for energy, sensing, and communication. iScience.

[10] Curteza A., Cretu V., Macovei L., Poboroniuc M. (2016). The manufacturing of textile products with incorporated electrodes. Autex Res. J..

[11] Euler L., Guo L., Persson N.-K. (2022). A review of textile-based electrodes developed for electrostimulation. Text. Res. J..

[12] Leon A.L., Manea L.R., Hristian L. (2016). Recent researches concerning the obtaining of functional textiles based on conductive yarns. IOP Conf. Ser. Mater. Sci. Eng..

[13] Gaubert V., Gidik H., Koncar V. (2020). Boxer underwear incorporating textile moisture sensor to prevent nocturnal enuresis. Sensors.

[14] Ismar E., Kurşun Bahadir S., Kalaoglu F., Koncar V. (2020). Futuristic clothes: Electronic textiles and wearable technologies. Glob. Chall..

[15] Gupta K.K., Abbas S.M., Abhyankar A.C. (2022). Effect of yarn composition and fabric weave design on microwave and EMI shielding properties of hybrid woven fabrics. J. Text. Inst..

[16] Pušić T., Saravanja B., Malarić K. (2021). Electromagnetic shielding properties of knitted fabric made from polyamide threads coated with silver. Materials.

[17] Sirková B.K., Tunáková V., Tunák M., Jezik K. (2022). Influence of woven fabric construction parameters on electromagnetic shielding effectiveness: Part I—Weave influence. Text. Res. J..

[18] Komolafe A., Torah R., Wei Y., Nunes-Matos H., Li M., Hardy D., Dias T., Tudor M., Beeby S. (2019). Integrating flexible filament circuits for e-textile applications. Adv. Mater. Technol..

[19] Jin H., Matsuhisa N., Lee S., Abbas M., Yokota T., Someya T. (2017). Enhancing the performance of stretchable conductors for e-textiles by controlled ink permeation. Adv. Mater..

[20] Hong H., Hu J., Yan X. (2020). Effect of the basic surface properties of woven lining fabric on printing precision and electrical performance of screen-printed conductive lines. Text. Res. J..

[21] Ojstršek A., Plohl O., Gorgieva S., Kurečič M., Jančič U., Hribernik S., Fakin D. (2021). Metallisation of textiles and protection of conductive layers: An overview of application techniques. Sensors.

[22] Pawlak R., Korzeniewska E., Koneczny C., Hałgas B. (2017). Properties of thin metal layers deposited on textile composites by using the PVD method for textronic applications. Autex Res. J..

[23] Tokarska M. (2019). Characterization of electro-conductive textile materials by its biaxial anisotropy coefficient and resistivity. J. Mater. Sci. Mater. Electron..

[24] Dušek K., Hrzina P., Tichy T., Reichl T., Vesely P., Dušek J. Evaluation of electrical properties of metalized woven and non-woven polymer-based textiles. Proceedings of the 43rd International Spring Seminar on Electronics Technology (ISSE).

[25] Wu X., Yu P., Sun X., Wang X., Li W. (2023). Modeling the thermal performance of anisotropic heat conduction fabric with different structural parameters. J. Nat. Fibers.

[26] Cappello L., Galloway K.C., Sanan S., Wagner D.A., Granberry R., Engelhardt S., Haufe F.L., Peisner J.D., Walsh C.J. (2018). Exploiting textile mechanical anisotropy for fabric-based pneumatic actuators. Soft Robot..

[27] Tokarska M., Gebremariam A., Puszkarz A.K. (2024). Assessment of the influence of fabric structure on their electro-conductive properties. Materials.

[28] Tokarska M., Miśkiewicz P., Pawlak W. (2023). Research on the planar electrical anisotropy of conductive woven fabrics. Adv. Eng. Mater..

[29] Van den Berg S., Luckabauer M., Wijskamp S., Akkerman R. (2023). Determination of the anisotropic electrical conductivity of carbon fabric reinforced composites by the six-probe method. J. Thermoplast. Compos. Mater..

[30] Liu Z., Xue Y., Sun B., Gu B., Hu M. (2024). Microstructural modeling on electrical conductivities of 3-D carbon/epoxy woven composites along different directions. Compos. Struct..

[31] Vasile S., Deruck F., Hertleer C., De Raeve A., Ellegiers T., De Mey G. (2017). Study of the contact resistance of interlaced stainless steel yarns embedded in hybrid woven fabrics. Autex Res. J..

[32] Xue Y., Li Z., Huang S., Xu X., Ding J., Gu B., Sun B. (2024). Micro contact modeling of electrical current conduction behavior between carbon fiber yarns. Compos. Sci. Technol..

[33] Tokarska M. (2022). A mixing model for describing electrical conductivity of a woven structure. Materials.

[34] Park J.B., Hwang T.K., Kim H.G., Doh Y.D. (2006). Experimental and numerical study of the electrical anisotropy in unidirectional carbon-fiber-reinforced polymer composites. Smart Mater. Struct..

[35] Hu J., Zhang X., Li G., Yang X., Ding X. (2016). Electrical properties of PPy-coated conductive fabrics for human joint motion monitoring. Autex Res. J..

[36] Hertleer C., Meul J., De Mey G., Vasile S.-I., Odhiambo S.A., Van Langenhove L. (2020). Mathematical model predicting the heat and power dissipated in an electro-conductive contact in a hybrid woven fabric. Autex Res. J..

[37] Tokarska M., Wróblewski P. (2024). Investigation of the contact resistance between stainless steel threads embedded in woven fabric. Indian. J. Fibre Text. Res..

[38] Timsit S. (1999). Electrical contact resistance: Properties of stationary interfaces. IEEE Trans. Compon. Packag. Technol..

[39] Gunnarsson E., Seoane F. (2023). Three-lead in vivo measurement method for determining the skin-electrode impedance of textile electrodes: A fast, accurate and easy-to-use measurement method suitable for characterization of textile electrodes. Text. Res. J..

[40] Shabani A., Hylli M., Kazani I., Berberi P., Zavalani O., Guxho G. (2019). The anisotropic structure of electroconductive leather studied by Van der Pauw method. Text. Leather Rev..

[41] Beldi M., Cayla A., Behary N., Perwuelz A. (2018). Effect of hybrid woven fabrics structure on their electrical properties. J. Mater. Sci. Mater. Electron..

[42] Angelova R.A. (2012). Determination of the pore size of woven structures through image analysis. Cent. Eur. J. Eng..

[43] Kostajnšek K., Zupin Ž., Hladnik A., Dimitrovski K. (2021). Optical assessment of porosity parameters in transparent woven fabrics. Polymers.

[44] Ragab A., Fouda A., El-Deeb H., Abou-Taleb H. (2017). Determination of pore size, porosity and pore size distribution of woven structures by image analysis techniques. J. Text. Sci. Eng..

[45] Mahltig B., Grethe T. (2022). High-performance and functional fiber materials-A review of properties, Scanning Electron Microscopy SEM and Electron Dispersive Spectroscopy EDS. Textiles.

[46] Karadag R., Oraltay R.G. (2023). Application of a new SEM-EDX technique associated with HPLC/DAD: Manufacturing methods of historical textiles. Archaeol. Anthropol. Sci..

[47] Skrzetuska E., Puszkarz A.K., Nosal J. (2024). Assessment of impact of the surface modification techniques on structural, biophysical, and electrically conductive properties of different fabrics. Materials.

[48] Jang J.U., Park H.C., Lee H.S., Khil M.S., Kim S.Y. (2018). Electrically and thermally conductive carbon fibre fabric reinforced polymer composites based on nanocarbons and an in-situ polymerizable cyclic oligoester. Sci. Rep..

[49] Sarkar K., Das D., Chaki T.K., Chattopadhyay S. (2017). Macro-structured carbon clusters for developing waterproof, breathable conductive cotton fabric. Carbon.

[50] Van der Pauw L.J. (1958). A method of measuring specific resistivity and Hall effect of discs of arbitrary shape. Philips Res. Repts..

[51] Renard M., Puszkarz A.K. (2022). Modeling of heat transfer through firefighters multilayer protective clothing using the computational fluid dynamics assisted by X-ray microtomography and thermography. Materials.

[52] Haupt F., Pult J.R.W., Hülsmann M. (2020). Micro-computed tomographic evaluation of the shaping ability of 3 reciprocating single-file nickel-titanium systems on single-and double-curved root canals. J. Endod..

[53] Griffiths A.J.V., Walther T. (2010). Quantification of carbon contamination under electron beam irradiation in a scanning transmission electron microscope and its suppression by plasma cleaning. J. Phys. Conf. Ser..

[54] Miśkiewicz P., Tokarska M., Frydrych I., Makówka M. (2021). Assessment of coating quality obtained on flame-retardant fabrics by a magnetron sputtering method. Materials.

[55] Corder G.W., Foreman D.I. (2009). Nonparametric Statistics for Non-Statisticians: A Step-by-Step Approach.

